# Autonomy Raises Productivity: An Experiment Measuring Neurophysiology

**DOI:** 10.3389/fpsyg.2020.00963

**Published:** 2020-05-15

**Authors:** Rebecca Johannsen, Paul J. Zak

**Affiliations:** ^1^Claremont Graduate University, Claremont, CA, United States; ^2^Center for Neuroeconomics Studies, Claremont, CA, United States

**Keywords:** organizations, decision-making, intrinsic motivation, behavior, experiment

## Abstract

Employees have been given increasing autonomy to work from home, from virtual offices, and during travel. Understanding why autonomy affects work behaviors has relied to date on self-reported data in which employees may consciously or unconsciously misattribute their own causal actions. We designed a neuroscience experiment to investigate the mechanisms through which greater autonomy affects individual and team performance and if this had an effect on mood. Participants (*N* = 100) were shown a three-min video that described the productivity impact of greater autonomy at work (treatment) or the productivity benefits of work-flow management software. Electrodermal responses were captured to measure physiologic effort and were related to the video stimuli, productivity, and mood. The treatment group had a 5.2% (*p* = 0.047) greater average productivity and 31% (*p* = 0.000) higher positive affect after the video than the control group average. Productivity was directly related to the physiologic effort put into the task for both the treatment and control groups, but the video prime did not increase effort compared to the control. The impact of physiologic effort on productivity continued to hold when controlling for participants’ intrinsic motivation. We also found that individual productivity was associated with an increase in positive affect, while group productivity increased positive affect only for those in the treatment group. Our findings indicate that increased perceived autonomy can significantly improve individual and group productivity and that this can have a salubrious impact on mood, but the neurologic mechanism through which this occurs remains to be identified.

## Introduction

Intangible rewards from work are important determinants of productivity and job satisfaction (Romaniuc, 2017). Due the difficulty of measuring intangible rewards, businesses traditionally rely on pay and benefit schemes to attract, motivate, and retain employees. Yet, pay is often fixed in the short- to medium-term and reliance on monetary incentives can decrease productivity (Frey and Jegen, 2001). Traditional labor economics predicts that employees will supply the minimum effort necessary to fulfill their stated duties, absent additional pay incentives to work harder (Lane, 1992; Spencer, 2003). Yet, there is abundant evidence that employees often expend substantial discretionary effort and that such effort is more common in some firms than in others (Lin, 2010; Rao et al., 2014). Beyond monetary compensation, most employees desire autonomy, purpose, and a sense of meaning from the work they do (Seppala and Cameron, 2015; Spencer, 2015; Zak, 2017b). These job characteristics are so important that people are willing to accept a lower salary for jobs that have autonomy and meaning (Hu and Hirsh, 2017) and may leave one organization for another that offers them these non-pecuniary benefits (Meister, 2012). Indeed, discretionary effort often depends on being recognized and rewarded for the additional effort, but these rewards do not have to be monetary (Zak, 2017b). Managers are tasked to use intangibles to increase motivation and productivity; however, few managers can design and consistently implement programs that influence discretionary effort (Baard et al., 2004; Chamorro-Premuzic and Garrad, 2017). Organizations with cultures that intentionally or unintentionally allow employees to obtain intangible rewards are successfully inspire discretionary effort and have improved business-relevant outcomes including lower job turnover and higher productivity (Long, 2012; Warrick, 2017).

Trust between work colleagues and supervisors motivates discretionary effort because it empowers colleagues to take ownership of their work, provide creative solutions, and fosters transparency from management (Seppala and Cameron, 2015; Brown et al., 2015; Zak, 2017b). Organizational trust has a number of constituent factors (Zak, 2017a, b). One of these is the locus of control over one’s work activities (we will use “empowerment” and “locus of control” interchangeably; Zak, 2017a). Employees who have the autonomy to execute projects as they see fit and/or to choose the projects they work on, are more productive and more satisfied with their jobs (Baard et al., 2004; Hogan and Coote, 2014; Wu et al., 2014; Zak, 2017b).

Autonomy for individuals as well as in teams increases ownership of outcomes and improves performance (Cordery et al., 2010; Zak, 2017b). Employees who have a high locus of control are able raise questions without adverse consequences and thereby improve output quality (Long, 2012). Hogan and Coote (2014) present evidence that innovation is higher when employees have autonomy due to greater persistence in overcoming problems for projects they control. Ceding control to employees is often feared by managers because it reduces the ease in which employees can be monitored and may increase the opportunity for shirking (Spencer, 2003; Arocena et al., 2010). However, when autonomy is given in an environment of organizational trust and combined with intrinsic motivation, employees exert discretionary effort rather than shirking (Thomas, 1990; Spencer, 2003; Baard et al., 2004; Wu et al., 2014; Zak, 2017b).

Productivity at work has been shown to improve well-being (Spencer, 2014, 2015; Hagler et al., 2016). This is due in part to improved positive mood that comes from satisfying work. A study of 12,000 employee diary entries showed that 76% of people’s best-mood days occurred when they were productive (Amabile and Kramer, 2011). Employees working in organizations that have a culture of trust, which includes high locus of control for employees, report happier than do employees low-trust organizations (Zak, 2017a, b).

In Zak (2017b), a component of organizational trust called “Yield,” captures the effect of organizational policies that give employees autonomy. This is presumed to improve productivity by increasing effort put into projects by employees. However, the mechanism through which autonomy improves performance is unknown. We hypothesized that empowering workers with autonomy would increase autonomic arousal as more effort would be put into executing projects. This will increase productivity, while at the same time resulting in improved mood.

## Materials and Methods

### Participants and Procedures

100 people were recruited (51 female, 49 male, Caucasian 40, African-American 7, Latino 15, Asian 35, Middle Eastern 1, and Other 2) for a study that lasted approximately 1 h. Participants were drawn from our existing study pool and were primarily made up of undergraduate and graduate students. Some members of the local community also participated. We did not require full-time work experience as a condition of inclusion. The experiment was run at the Center for Neuroeconomics Studies at Claremont Graduate University exempted by the Institutional Review Board of Claremont Graduate University (IRB # 2922). All participants gave written informed consent prior to inclusion. Individuals in the study ranged from 18 to 69 years old (*M* = 27, SD = 10.92) and were randomly assigned to the control or treatment groups; each condition had 50 participants.

**FIGURE 1:**
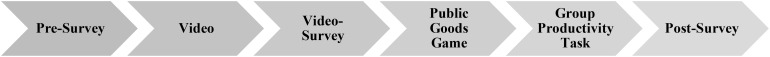
The time course of the experiment.

Figure 1 shows the timeline of the study in which participants answered surveys, watched a 3-min video, made a contribution decision, and worked as a team to solve math problems. The surveys collected information about demographics, motivation (Intrinsic Motivation Inventory, IMI; Deci and Ryan, 2000), mood (Positive and Negative Affect Scale, PANAS; Watson et al., 1988), and closeness to others (Inclusion of Others in the Self scale, IOS; Aron et al., 1992). Analysis of participant demographics verified an equal distribution between treatment and control conditions. Changes in the PANAS and IOS were analyzed to understand the subjective psychological responses to the stimuli.

Participants were recruited in groups of four for each session of the experiment, with the exception of four groups of three participants (two in each condition). Prior to the behavioral tasks, participants watched a three-min video as a group. Treatment participants watched an animated white-board video that one of the authors wrote and narrated (PJZ). The video discussed how work colleagues can be empowered with autonomy to be more successful team members and is an excerpt from Zak (2017b). The video was included to test the hypothesis that influencing participants perceived locus of control, would increase effort toward group goals. The control group watched a video in the same style as the treatment stimulus that discussed how work-flow software could be used to increase productivity. Transcripts of both videos are included in the section “Appendix.”

Next, participants made decisions in the Public Goods Game (PGG). This measures monetary contributed toward a group goal (Lang et al., 2018). Participants made choices in two rounds of the PGG and were endowed with $4 for each round. They were instructed to contribute some, all, or none of their endowment to a common pool. Funds contributed would be doubled and evenly distributed to group members. Participants were not shown the results from this task until their final payout at the end of the study and were therefore unaware of choices made by other group members.

The second behavioral task was designed for this study to measure team productivity. Participants were seated in chairs around a table and were given math problems to solve as a team. Groups of four were given five sheets of two-digit addition problems and had 3 min to answer as many correctly as possible. If they answered 75% or more of the math problems correctly, each participant would be paid $10, and otherwise they would each earn nothing. Prior to starting, participants were given 2 min to develop a strategy. At the end of the discussion period, worksheets were handed to the team leader (chosen randomly as the person who sat in the seat closest to a window) and the timer was started. At the end of 3 min, sheets were collected and graded in private while participants completed post-task surveys. Groups with three members were given four worksheets and had otherwise identical procedures. This task is designed to quantify the ability of teams to organize rapidly to accomplish a goal.

Earnings were privately paid for both the contribution and productivity tasks and participants were dismissed. Individuals earned between $16 and $30 each depending on their choices and choices made by those in their group.

### Causal Model

**FIGURE 2:**
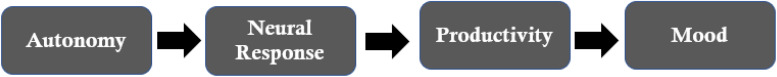
The causal model the experiment will test.

Figure 2 presents a schematic model that identifies the causal relationships we will test. We hypothesized that the video stimuli would produce a neurologic response in participants that would result in a change in their psychological states that would affect productivity and this would affect mood. To establish causation, we are providing a treatment stimulus designed to increase locus of control, empowering participants to take ownership over their work and provide creative solutions. Outcomes will be compared between the treatment and control groups to establish size effects and predictive accuracy.

### Physiology

Autonomic arousal can be measured with electrodermal activity (EDA). EDA captures that change in electrical resistance from palmar sweat (Figner and Murphy, 2011; Braithwaite et al., 2015). The primary measures of EDA are skin conductance levels (SCL) and skin conductance responses (SCRs). SCL is the tonic or continuously changing conductance of the skin, while SCR captures phasic or peak responses to stimuli (Figner and Murphy, 2011). Both SCL and SCR measure sympathetic arousal during an experience (Braithwaite et al., 2015) and are widely used because they link neurologic activity to psychological states and behaviors (Kramer, 2007; Jasniewski et al., 2017).

Cardiac and EDA were collected using a Biopac MP150 data acquisition system (Biopac Inc, Goleta, CA, United States). Data were visually inspected in AcqKnowledge software version 4.2 (Biopac Inc., Goleta, CA, United States), transformed and extracted for the baseline and video periods. Both EDA measures were baseline corrected prior to analysis. Skin conductance waveforms with signal loss and data drop-offs shorter than 1 s were replaced with averages from adjacent parts of the waveform. To remove high-frequency noise and skew, a 10-Hz low-pass filter (Norris et al., 2007), and a square root transformation (Dawson et al., 1989; Figner and Murphy, 2011) were applied. Non-specific skin conductance responses (NS-SCRs) were identified using a threshold of 0.01 μS.

### Variables

The dependent variables are the accuracy on the productivity task (individual and group) and PGG contributions. Productivity for individuals and groups is measured as the sum of the two-digit addition problems. The behavioral measure of cooperation, the PGG, is measured as the amount of money contributed to the common pool in round 1 and round 2 of this task.

We did not want to influence participants by asking survey questions about their perceived locus of control, rather, we used the treatment video to seek to influence participants’ perceptions of their ability to control how they accomplished tasks. We included a measure of group closeness to test whether individual variations in how people conform in groups affects productivity (Reagans and Zuckerman, 2001; Zak, 2017b). Additional control variables include intrinsic motivation (Becchetti et al., 2013), gender, age, income, education, work hours, and GPA.

The causal model posits that productivity affects mood following previous findings (Amabile and Kramer, 2011). Yet, there are some reports of in mood increasing productivity (Kaufmann, 2003; Hagler et al., 2016). Our experimental approach measures the change in mood from baseline twice, after the video prime and after the productivity task in order to provide insights into the role of mood at work.

### Analysis

Data analysis was performed using Pearson’s χ^2^, Wilcoxon differences in means, Spearman correlations, and least squares regressions. These analytics were used because the Shapiro–Wilk test indicated that productivity, PGG contributions, mood, closeness, intrinsic motivation, and SCL were not normally distributed (see section “Appendix”). In addition to ordinary least squares, we estimated regressions using non-linear parameters, and logarithmic transformations to verify predictive accuracy and size effects.

## Results

Demographic variables were not statistically different between the control and treatment groups (ps > 0.13) nor were they significantly correlated with the dependent variables (ps > 0.24. They were therefore not included in the analyses.

### Behavior

#### Productivity

**FIGURE 3:**
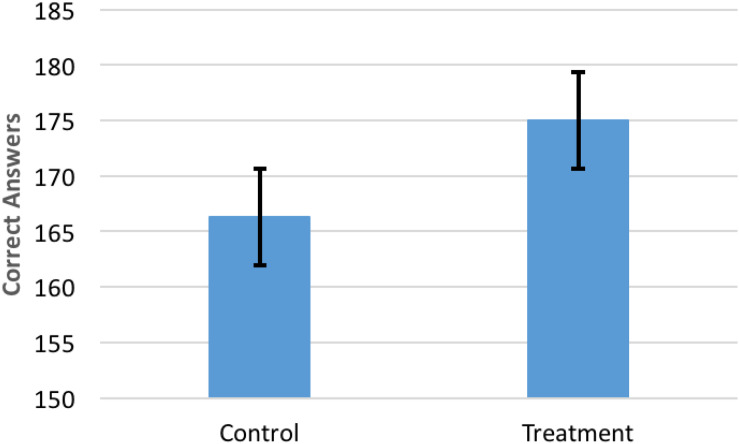
The autonomy video increased productivity by 5.2% (*p* = 0.047) compared to the control video. Groups did not differ in demographics. Bars shown are standard errors.

All groups in the treatment and control conditions passed the minimum threshold of accuracy for the productivity task and earned the $10 incentive. The autonomy stimulus significantly increased average productivity; the treatment group correctly answered 9 more questions, outperforming the control group by 5.2% [χ^2^(20) = 72, *p* = 0.000; Figure 3]. There are 8 observations for treatment group productivity that are 1.5 standard deviations below the mean. If these are dropped from the analysis, treatment productivity is higher, 5.4% (*p* = 0.01). All subsequent analyses include these outliers providing conservative estimates for the treatment. Individual productivity was not different between conditions [χ^2^(42) = 42.34, *p* = 0.456].

#### Public Good Game

Contributions in the PGG were not statistically different between the treatment and control groups, nor was there a difference in amounts contributed per round (ps > 0.12). Participants in the treatment group contributed an average of $2.74 in the first round of the PGG compared to $2.82 contributed by the control group (*t* = −0.33, *p* = 0.746, and *d* = 0.065). The second round was similar with $2.40 contributed on average by the treatment group and $2.70 by the control group (*t* = −1.06, *p* = 0.292, and *d* = 0.212). Averaging both rounds, contribution amounts were not statistically different between the treatment and control groups [χ^2^(17) = 17.75, *p* = 0.405].

#### Physiology

The treatment and control videos increased SCL from baseline (*T*: *M* = 9.1%. SD = 5.8%, *p* = 0.000; *C*: *M* = 12.9%, SD = 10.7%, and *p* = 0.0000) and had a significant difference by condition (Wilcoxon *z* = 2.69, *p* = 0.0072). Consistent with our hypothesis, the change in SCL was positively associated with both individual productivity (β = 31.09, *t* = 2.38, *d* = −5.32, *p* = 0.01, and one-tailed test), and group productivity (β = 57.69, *t* = 2.03, *d* = −10.66, *p* = 0.0225, and one-tailed test) in an ordinary least squares regression that includes a condition indicator.

The analysis also showed that SCL was positively correlated with PGG contributions in the second round (*r* = 0.20, *p* = 0.03, and one-tailed test), but not with contributions in the first round (*r* = 0.14, *p* = 0.102, and one-tailed test). SCL was not correlated with the change in positive affect at the end of the study (*r* = −0.19, *p* = 0.06) and SCR was not associated with individual (*p* = 0.53) or group productivity (*p* = 0.80).

#### Mood

The treatment video increased positive affect by 31% (Wilcoxon test statistic *z* = −4.24, *p* = 0.000, and *d* = −0.902) and left negative affect unchanged (*z* = −0.71, *p* = 0.477, *d* = −0.124). But, the change in positive affect from the video was not correlated with group or individual productivity (ps > 0.32). As hypothesized in the causal model, productivity was correlated with the change in positive affect from baseline at the end of the study. This holds for individual productivity for the treatment and control groups (*T*: *r* = 0.35, *p* = 0.0122; *C*: *r* = 0.30, *p* = 0.0336) and team productivity for the treatment group but not the control group (*T*: *r* = 0.26, *p* = 0.036; *C*: *r* = −0.11, *p* = 0.446). Neither positive nor negative affect was associated with contributions in either round of the PGG (ps > 0.626).

#### Intrinsic Motivation and Closeness

Intrinsic motivation was positively correlated with individual productivity (*r* = 0.49, *p* = 0.002), for both the treatment (*r* = 0.50, *p* = 0.001) and control groups (*r* = 0.30, *p* = 0.035). For example, participants in the top third of intrinsic motivation were 17.5% (*t* = 2.58, *p* = 0.011, and *d* = 5.37) more productive than those in the lowest third, answering 7 more questions correctly on average. Intrinsic motivation was not significantly correlated with group productivity or PGG contributions (ps > 0.85), nor did it vary between treatment and control groups [χ^2^ (19) = 14.28, *p* = 0.767].

Intrinsic motivation was associated with the change in mood during the video (*r* = 0.33, *p* = 0.0012), for both the treatment group and control groups (*T*: *r* = 0.41, *p* = 0.005; *C*: *r* = 0.25, *p* = 0.079). Intrinsic motivation was also correlated with change in positive affect at the end of the study (*r* = 0.45, *p* = 0.000) for both treatment and control groups (*T*: *r* = 0.43, *p* = 0.002; *C*: *r* = 0.49, *p* = 0.0002).

While there was no difference in average closeness between treatment and control groups [χ^2^(18) = 21.1, *p* = 0.274], closeness was positively correlated with individual productivity (*r* = 0.012, *p* = 0.091). Closeness was not significantly correlated with group productivity or PGG contributions (ps > 0.59).

#### Overall Effects

The autonomy prime and SCL increased individual and group productivity (ps < 0.05) when controlling for intrinsic motivation (equations 1–2, Table 1). Including the additional control group closeness results in SCL affecting individual but not group productivity while the treatment only affects group productivity (equations 3–4, Table 1). The causal relations in Figure 2 are further tested by investigating the mediating effects of SCL on individual and team performance using a path analytic model. For team performance the direct effects of both the autonomy prime and SCL were statistically significant [Autonomy: *t* = 2.38, *p* = 0.017, *d* = 0.402; SCL: *t* = −2.08, *p* = 0.037), *d* = 10.66], but the indirect effect was not (*t* = −1.49, *p* = 0.137]. Estimating the model for individual productivity shows that SCL directly impacted individual performance, but the autonomy prime and indirect effects were not significant (Autonomy: *t* = 1.35, *p* = 0.176, *d* = 0.402; SCL: *t* = −2.10, *p* = 0.036, *d* = 10.66; and Indirect: *t* = −1.49, *p* = 0.135).

**TABLE 1:** The autonomy treatment and SCL increase individual and team productivity when intrinsic motivation is included as a control.

	(1)	(2)	(4)	(3)
Variables	Individual productivity	Group productivity	Individual productivity	Group productivity
SCL	31.09**	57.69*	31.77*	44.26
	(13.08)	(28.40)	(13.87)	(29.38)
Autonomy prime	3.943*	11.48*	3.629	10.91*
	(2.283)	(4.959)	(2.386)	(5.056)
Intrinsic motivation	0.899**	−0.0229	0.941**	−0.0379
	(0.262)	(0.568)	(0.273)	(0.579)
Closeness			−0.00563	0.479
			(0.626)	(1.326)
Constant	28.16**	158.9**	27.94**	160.1**
	(4.002)	(8.691)	(4.526)	(9.591)
Observations	88	88	85	85
*F*-statistic	5.83	2.62	4.34	1.43
*p*-value	0.001	0.056	0.003	0.23
*R*-squared	0.172	0.086	0.178	0.067

With both intrinsic motivation and closeness as controls, SCL increases individual but not group productivity while the treatment only affects group productivity. Standard errors in parentheses. **p < 0.01, *p < 0.05 one-tailed *t*-tests.

## Discussion

The autonomy video was designed to increase team productivity through the Yield component of organizational trust. While survey data have shown that employee autonomy increases productivity (Zak, 2017b), the behavioral neuroscience experiment reported here sought to identify why employees empowered with autonomy are more productive. The analysis showed that both videos caused an increase in SCL from baseline and that there was a linear relationship between the change in SCL and individual and team productivity. The increased arousal improved team outcomes through increased effort toward a group goal.

The change in individual productivity had a positive impact on change in mood from baseline to the post-work period (*t* = 38.9, *p* = 0.000, and *d* = 5.5). The mood change in response to the video was unrelated to productivity. This supports prior findings showing that productivity increases positive affect rather than the converse (Lyubomirsky et al., 2005; Zhai et al., 2009; Amabile and Kramer, 2011). The impact of effort on individual productivity was enhanced by intrinsic motivation (*t* = 25.7, *p* = 0.000, and *d* = 3.78). The stated purpose of successfully completing the math task was to earn $10, yet, participants who were intrinsically motivated had greater productivity and improved mood despite earning the same amount as others for this task (*r* = 0.49, *p* = 0.002; *r* = 0.45, *p* = 0.000).

Although our neurophysiologic measure of effort, SCL, impacted individual and group productivity, the mediating effects of SCL on productivity were not significant. This indicates that the causal model in Figure 2 should be modified so that autonomy has a direct impact on productivity. SCL and intrinsic motivation are better understood as measures of effort. Specifically, SCL can be considered the acute response to the experimental task while intrinsic motivation is a trait response. One contribution of the study reported here is that both the state and trait effort affect productivity.

The autonomy video increased positive affect by 31% (*z* = −4.24, *p* = 0.000, and *d* = −0.902) but higher SCL reduced positive affect (*r* = −0.20, *p* = 0.014). Work is effortful, and greater work effort was less enjoyable to participants. Work effort appears to have generated camaraderie among participants with change in SCL associated with increased closeness to one’s work group (*r* = 0.207, *p* = 0.015). While closeness was associated with individual productivity (*r* = 0.18, *p* = 0.082), it was not correlated with team productivity or PGG contributions.

Our findings offer physiologic support for the JCDS (Job Control Discretion Support) model (Johnson and Hall, 1988; Karasek, 1979). In addition to autonomy, research on the JCDS model has shown how support increases productivity and the well-being of employees (Häusser et al., 2010). Low locus of control at work coupled with high job demands rapidly diminish performance. Our results cannot rule out an inverted-U relationship between arousal and performance. Previous findings report that insufficient arousal and extreme arousal reduce job performance following the Yerkes-Dodson law (Anderson, 1994; Gino, 2016). Greater autonomy, more social support, or reduced job demands can keep employees in the middle-arousal zone where performance and well-being are both high (Kim and Stoner, 2008; Fernet et al., 2013). These effects appear to be particularly acute in older workers (Giorgi et al., 2020). Future research can supplement the protocol in the present experiment with stressors to identify if these reduce task performance. Indeed, measuring SCL is an effective way to measure the objective arousal of stressors.

Our results show the subtlety of the relationship between autonomy, mood, and productivity. Videos, and potentially other interventions meant to increase employees’ locus of control at work, may improve mood and closeness to work colleagues. Although it is unclear from this study how these psychological states impact organizational goals, mood is directly linked with business-relevant outcomes and employee well-being (Hu et al., 2017; Lavy and Littman-Ovadia, 2017; Penalver et al., 2019). We are left with a dilemma for managers who seek to motivate higher productivity via discretionary effort. Effort appears to build team ties, improve individual and team productivity but had an ambiguous effect on mood and closeness.

Interestingly, participant earnings were not correlated with performance or mood (ps > 0.27), suggesting that autonomy, rather than pay, motivated the increase in productivity, and positive mood. This suggests that intangible elements, such as autonomy, were the main motivating factors for the changes in productivity. With global competition and slimming profit margins, this is good news for organizations. This doesn’t mean pay is irrelevant. Once a fair wage is offered, organizations have options for improving relevant outcomes for themselves and their employees without needing to substantially increase their costs.

The present study faces several limitations. These include using a primarily student sample, testing in a laboratory rather than in a workplace, a task that was only moderately demanding, and a congenial atmosphere during testing. The laboratory environment facilitated the measurement of EDA, but inhibits the generalizability of our findings. We did not perform an *ex ante* sample size calculation although previous studies using peripheral neural measures show that *N* = 100 is sufficient to reach a power of test of 0.99 (Alexander et al., 2018). While participants were randomized into the control and treatment groups and testing showed statistically identical demographics, we cannot rule out unmeasured confounds that might drive the results. In addition, the measure of productivity used in the study does not capture the variations in work product across industries, for example, in manufacturing.

## Conclusion

The present study posited a neurologic mechanism through by which autonomy would affect team productivity. A video prime for autonomy increased productivity by 5.2% but this was not due to additional physiologic effort as measured by the EDA response. At the same time, physiologic effort linearly increased both individual and team productivity. We find only partial support for the schematic causal model linking autonomy to productivity. Further research assessing neurophysiologic responses during work tasks is warranted to understand the mechanism through which autonomy influences productivity. A contribution of this study is to demonstrate that measuring physiologic responses provide insights that complement previous findings using self-reports.

Our findings demonstrate that perceived, rather than actual, autonomy impacts effort and work output. Workplaces that seek to apply such an intervention should increase actual rather than perceived autonomy in order to achieve more than transitory productivity gains. Understanding autonomy is particularly important with the rise of telecommuting and geographically-separated work groups. Our lab is currently testing autonomy interventions in field studies to assess whether the laboratory results reported here generate improvements in productivity. Knowing that a laboratory autonomy intervention increases productivity is the first step toward endowing employees with more control over what they do at work.

## Data Availability Statement

The datasets generated for this study are available on request to the corresponding author.

## Ethics Statement

The studies involving human participants were reviewed and approved by Claremont Graduate University’s IRB. The patients/participants provided their written informed consent to participate in this study.

## Author Contributions

PZ designed, funded, contributed to analysis, and co-wrote the manuscript. RJ ran study, led analysis, and co-wrote the manuscript.

## Conflict of Interest

The authors declare that the research was conducted in the absence of any commercial or financial relationships that could be construed as a potential conflict of interest.

## Appendix

### Treatment Video Transcript

Often younger and less experience colleagues will be your chief innovators. You know how many dumb things you did when you were young, but some of these paid off. Then when you were older and wiser you made fewer positive and negative deviations from what is expected. Empower younger colleagues to experiment because the experts do not always have the most innovative ideas. Case and point. In 2004 the US congress mandated that by 2015 1/3 of military ground vehicles had to be autonomous. Initially, established automobile manufacturers were funded to produce autonomous vehicles. After 5 years and significant investment by the United States government, no progress had been made. Changing tack, the Defense Advanced Research Projects Agency (DARPA) offered all comers a $1 million prize for a self-driving car that could complete a course in the Mojave Desert in less than 10 h. Just 2 years later a group of engineering students from Stanford University won the challenge. In a similar vein, in 2012 a pair of University of Toronto graduate students were the first in the world to produce sustained flight in a human-powered helicopter. Established engineers were convinced a human-powered helicopter wasn’t possible. These young people broke rules they did not even know existed to create spectacular breakthroughs. Incremental improvements are made when one uses single-loop learning. This approach improves a process or product by refining existing techniques. Radical improvements come from double-loop learning in which the underlying assumptions about the mechanisms producing results are questioned and sometimes discarded. Double-loop learning even questions the reasons why an innovation is needed. Asking, “why did this happen rather than how do we fix this?” Young people who are less wedded to tradition may be better at double loop learning and thus at creating significant innovations. Yield provides the space for double-loop learning. High-trust cultures permit core business processes to be challenged even if they have worked well in the past or were established by the founders. You can implement Yield by using objective data to determine improvements and disseminating findings organization-wide. What’s right, is right. Colleagues can tweak the existing operating model, or they can throw the model out and try to materially accelerate performance. The latter is more likely when Yield is high.

**TABLE A1:** Shapiro–Wilk test for normality.

	***W***	***V***	***Z***	**Prob > *z***
Individual productivity	0.93718	5.186	3.652	0.0001
Group productivity	0.89132	8.973	4.868	0.0000
PGG round 1	0.96634	2.779	2.267	0.0117
PGG round 2	0.98081	1.584	1.021	0.1537
PGG combined	0.97133	2.367	1.912	0.0280
IOS	0.88231	9.473	4.98	0.0000
PANAS pos	0.87091	10.658	5.249	0.0000
PANAS neg	0.96356	3.009	2.444	0.0073
Intrinsic motivation	0.96879	2.577	2.1	0.0179
RR	0.95459	3.624	2.85	0.0022
SCL	0.84561	11.463	5.373	0.0000

### Control Video Transcript

In every industrial setting there is a stream of critical operational events. These events need to be documented along with the actions performed by the operator in order for any issues to be effectively tracked, dealt with, and communicated to all other plant users. The recording and tracking of these events is mandatory in many plants and quick and efficient retrieval of information is essential to save time and enable plant optimization. Many companies who use paper log books to record event details are now converting from paper to softcopy, using J5 to increase their efficiency. The J5 operations logbook allows users to easily and accurately record event data and follow-up actions through its intuitive browser-based interface. Electronic operator logs are categorized by their onsite location and type. Here users can provide all necessary details of an event, including the date and time in which the event occurred and the current status and prxiority. All logs will also include the names of the user responsible for creating and modifying the logs. Since operations logs are categorized by their onsite location, any new log for a particular area is made visible to all other users in that same area. Some users may also be granted additional rights to modify, close or cancel logs made visible to them. A log is owned by the user who created it and will remain open until the owner closes it. Once a log has been closed, it cannot be reopened and will remain saved in the J5 system for archiving purposes. Logs in Operations Logbook have a notes tab that can be used by all to provide additional information or comments to any selected log. The name of the user responsible for its creation, as well as the date and the time that the note was added are recorded on the note, allowing users to follow-up with the person responsible on any notes that may not be clear. At the end of the shift all operations data is collated, and important entries are attached to the handover and presented in a handover report for management in the oncoming shift, providing overall communication on the plant.
